# Genetical Genomics: Spotlight on QTL Hotspots

**DOI:** 10.1371/journal.pgen.1000232

**Published:** 2008-10-24

**Authors:** Rainer Breitling, Yang Li, Bruno M. Tesson, Jingyuan Fu, Chunlei Wu, Tim Wiltshire, Alice Gerrits, Leonid V. Bystrykh, Gerald de Haan, Andrew I. Su, Ritsert C. Jansen

**Affiliations:** 1 Groningen Bioinformatics Centre, Groningen Biomolecular Sciences and Biotechnology Institute, University of Groningen, Kerklaan 30, Haren, The Netherlands; 2 Department of Human Genetics, Medical Center Groningen, University of Groningen, Groningen, The Netherlands; 3 Genomics Institute of the Novartis Research Foundation, San Diego, California, United States of America; 4 School of Pharmacy, University of North Carolina, Chapel Hill, North Carolina, United States of America; 5 Department of Cell Biology, University Medical Center Groningen, University of Groningen, Groningen, The Netherlands; Jackson Laboratory, United States of America

Genetical genomics aims at identifying quantitative trait loci (QTLs) for molecular traits such as gene expression or protein levels (eQTL and pQTL, respectively). One of the central concepts in genetical genomics is the existence of hotspots [1], where a single polymorphism leads to widespread downstream changes in the expression of distant genes, which are all mapping to the same genomic locus. Several groups have hypothesized that many genetic polymorphisms—e.g., in major regulators or transcription factors—would lead to large and consistent biological effects that would be visible as eQTL hotspots.

Rather surprisingly, however, there have been only very few verified hotspots in published genetical genomics studies to date. In contrast to local eQTLs, which coincide with the position of the gene and are presumably acting in *cis*—e.g., by polymorphisms in the promoter region—distant eQTLs have been found to be more elusive. They seem to show smaller effect sizes and are less consistent, perhaps due to the indirect regulation mechanism, resulting in lower statistical power to detect them and, consequently, an inability to reliably delimit hotspots [2]. While there are typically hundreds to thousands of strong local eQTLs per study, the number of associated hotspots is much lower. For example, a recent very large association study in about 1,000 humans did not find a single significant hotspot [3]. Other studies have reported up to about 30 hotspots, far less than the number of significant local eQTLs (Table 1). The molecular basis is known for less than a handful of cases. An example is the *Arabidopsis* ERECTA locus, which leads to a drastic phenotypic change in the plant and has broad pleiotropic effects on many molecular (and morphological) traits [4].

**Table 1:** eQTL Hotspots Reported in Selected Genetical Genomics Studies.

Paper	Organism	Population Size	Number of Local eQTLs	Number of Distant eQTLs	Threshold for eQTLs	Number of Hotspots
Brem et al., Science, 2002 [23]	yeast	40	185	385	*p*<5×10^−5^	8
Yvert et al., Nat Genet, 2003 [13]	yeast	86	578	1,716	*p*<3.4×10^−5^	13
Schadt et al., Nature, 2003 [1]	mouse	111	1,022	1,985	LOD>4.3	7
Kirst et al., Plant Physiol, 2004 [24]	eucalyptus	91	1	8	experiment-wise α = 0.10	2
Monks et al., AJHG, 2004 [25]	human	15 CEPH families (167)	13	20	*p*<5×10^−5^	0
Morley et al., Nature, 2004 [26]	human	14 CEPH families	29	118	*p*<4.3×10^−7^	2
Cheung et al., Nature, 2005 [27]	human	57	65	0	*p*<0.001	0
Stranger et al., PLoS Genet, 2005 [28]	human	60	10–40	3	corrected *p*-value = 0.05	0
Chesler et al., Nat Genet, 2005 [29]	mouse	35	83	5	FDR = 0.05	7
Bystrykh et al., Nat Genet, 2005 [30]	mouse	30	478	136	genome-wide *p*<0.005	“multiple”
Hubner et al., Nat Genet, 2005 [31]	rat	259	622	1,211	*p*<0.05	2
Mehrabian et al., Nat Genet, 2005 [32]	mouse	111	20,107 total	20,107 total	LOD>2	1
DeCook et al., Genetics, 2006 [33]	*Arabidopsis*	30	3,525 total	3,525 total	FDR = 2.3%	5
Lan et al., PLoS Genet, 2006 [34]	mouse	60	723	5,293	LOD>3.4	15
Wang et al., PLoS Genet, 2006 [35]	mouse	312	2,118	4,556	*p*<5×10^−5^	7
Li et al., PLoS Genet, 2006 [36]	*C. elegans*	80	414	308	*p*<0.001; FDR = 0.04	1
Keurentjes et al., PNAS, 2007 [4]	*Arabidopsis*	160	1,875	1,958	FDR = 0.05	∼29
McClurg et al., Genetics, 2007 [37]	mouse	32	N.A.	N.A.	N.A.	25
Emilsson et al., Nature, 2008 [3]	human	470	1,970	52	FDR = 0.05	0
Schadt et al., PLoS Biol, 2008 [38]	human	427	3,210	242	*p*<1.6×10^−12^	23
Ghazalpour et al., PLoS Genet, 2008 [39]	mouse	110	471	701	FDR = 0.1	4
Wu et al., PLoS Genet, 2008 [5]	mouse	28	600	885,840 (C. Wu and A. I. Su, unpublished data)	*p*<0.003	1,659

The numbers are based on the statistical procedure and threshold used in the original publication, which can vary widely between papers. Where results based on multiple thresholds were reported, we included the most conservative one in the table.

N.A., not reported in the original paper. FDR, false discovery rate.

Recently, Wu et al. [5] reported the large-scale identification of hotspots. They studied gene expression in adipose tissue of 28 inbred mouse strains and performed eQTL analysis by genome-wide association analysis. The paper reports the identification of over 1,600 candidate hotspots, each with a minimum hotspot size of 50 target genes. Furthermore, they demonstrated that these hotspots are biologically coherent by showing that in about 25% of cases, the hotspot targets are enriched for functional gene sets derived from Gene Ontology, the KEGG pathways database, and the Ingenuity Pathways Knowledge Base. These findings suggested that genetic polymorphisms can indeed lead to large and consistent biological effects that are visible as eQTL hotspots.

However, the authors chose a relatively permissive threshold of *p* = 0.003 for QTL detection, uncorrected for multiple testing. In total, 886,440 eQTLs were identified at this threshold, i.e., 134 per gene. A permutation test (C. Wu and A. I. Su, unpublished data) shows that this results in a false discovery rate of 64%, largely resulting from multiple testing across 157,000 SNPs and 6,601 probe sets. This relatively permissive threshold was chosen because the focus of the analysis was on patterns of eQTL hotspots and not on individual eQTL associations. Analysis of eQTL patterns is relatively robust to individual false positives, and a permissive threshold allows for relatively greater sensitivity in detecting signal [6]. The authors observed an enrichment of specific biological functions among the genes in the reported hotspots. The study also reported that enriched categories tended to match the annotation of candidate regulators. Moreover, one predicted regulator was experimentally validated. In sum, these data seem to support the hypothesis that hotspots are downstream of a common master regulator linked to the eQTL.

However, we suggest here that these observations may also be explained by clusters of genes with highly correlated expression. If one gene shows a spurious eQTL, many correlated genes will show the same spurious eQTL, in particular if the false discovery rate for individual eQTLs is very high [2], [7]–[9]. There are many nongenetic mechanisms that can create strongly correlated clusters of functionally related genes. On the one hand, such clusters may be a result of a concerted response to some uncontrolled environmental factor. On the other hand, dissected tissue samples can contain slightly varying fractions of individual cell types, leading to cell-type–specific gene clusters, which vary in a correlated manner. The resulting correlation patterns represent potentially confounding effects, both for the correct determination of a significance threshold and for the biological interpretation of the resulting hotspots.

**Figure 1:**
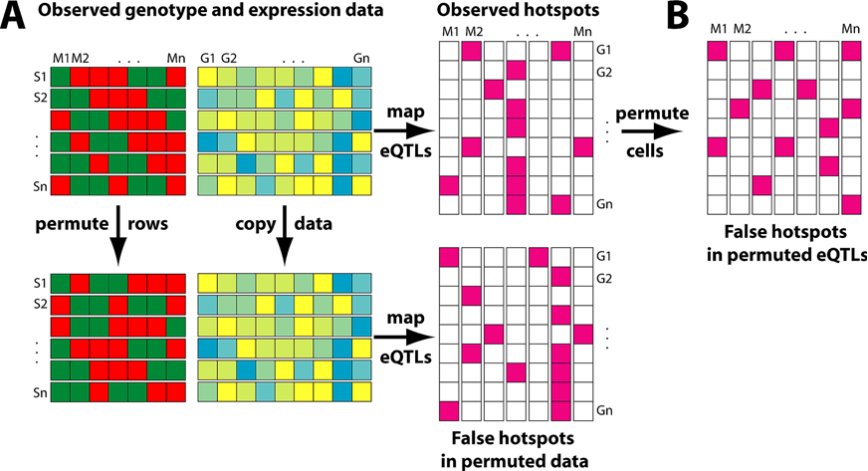
Alternative Permutation Strategies for Determining the Significance of eQTL Hotspots in Linkage and Association Studies. (A) The top panel shows the original data. The genotype matrix contains information about the genotype of each strain (S_1_…S*_n_*) at each marker position along the genome (M_1_…M*_n_*). For each strain, the expression of genes G_1_…G*_n_* is measured. Linkage or association mapping combines these two sources of information to yield the eQTL matrix, where each purple entry indicates a significant linkage or association for a gene at a particular locus. The bottom panel illustrates the permutation strategy advocated here, where the strain labels are permuted, so that each strain is assigned the genotype vector of another random strain, while the expression matrix is unchanged. When the mapping is repeated on these permuted data, the correlation structure of gene expression is maintained, leading to an accurate estimate of the clustered distribution of false eQTLs along the genome. (B) shows the permutation strategy used in [5], where the original eQTL matrix is permuted by assigning the same number of eQTLs to genes randomly. The correlation of gene expression is lost, leading to an underestimate of the clustered pattern of spurious eQTLs.

Consequently, a key consideration in eQTL analysis is in the effective design of a permutation strategy to assess statistical significance. The approach used in [5] permuted the observed eQTLs among genes (Figure 1B). However, this approach has the disadvantage of ignoring the expression correlation between genes so that their spurious eQTLs no longer cluster along the genome. This permutation strategy leads to a potentially severe underestimate of the null distribution of the size of hotspots, when there are correlated clusters as described above.

An alternative strategy would have been to permute the strain labels as shown in Figure 1A, maintaining the correlation of the expression traits while destroying any genetic association [2],[10]. As discussed above, it is expected that this would result in a more realistic significance threshold and a much smaller number of significant hotspots. Reanalysis of the data from [5] confirmed this idea: when permuting the strain labels (i.e., randomly swapping the genotypes between animals), the average maximum size of hotspots in the permuted data increases from less than 50 to 986. Consequently, even the largest hotspot in the real data only has a multiple testing corrected *p*-value of 0.23. This reanalysis demonstrates that expression correlation can indeed explain a large part of the co-mapping between genes. Such effects may also underlie some of the higher numbers of hotspots reported by some earlier studies (Table 1), especially where no appropriate permutation tests were applied to determine the statistical significance of hotspots [2].

Of course, this does not imply that all hotspots are necessarily false positives. As described above, about 5% of the co-mapping clusters in [5] are not only functionally coherent but also map to a locus that contains a gene of the same functional class. This number is not statistically significant, but it is still suggestive of an enrichment of functional associations (*p*<0.16, false discovery rate = 67%; C. Wu and A. I. Su, unpublished data). Some of these prioritized hotspots could correspond to true hotspots, and indeed one of them has been verified experimentally: cyclin H was validated as a new upstream regulator of cellular oxidative phosphorylation, as well as a transcriptional regulator of genes composing a hotspot [5].

Other studies, which used much stricter thresholds for defining their hotspots, also demonstrated the potential of interpreting putative hotspots by a closer study of the associated genetic locus [11],[12]. An example is the recent work of Zhu et al. [12]: by combining eQTL information, transcription factor binding sites, and protein–protein interaction data in a Bayesian network approach, they were able to predict causal regulators for nine out of the 13 hotspots (69%) originally reported in [13]. With integrated methods like these, it should be possible to identify those hotspots that are more than just clusters of co-expressed genes. As a result, the number of identified, functionally relevant hotspots could ultimately increase beyond the small numbers reported in Table 1. This would create new opportunities for gene regulatory network reconstruction.

In any case, for the time being it seems that distant eQTLs and their hotspots are still scarce and hard to find, and that those that are reported should be interpreted with caution. This rarity of convincing hotspots in genetical genomics studies is intriguing. It could be due to the limited power of the initial studies, but it could also have a more profound reason. For example, it might well be that biological systems are so robust against subtle genetic perturbations that the majority of heritable gene expression variation is effectively “buffered” and does not lead to downstream effects on other genes, protein, metabolites, or phenotypes [14]–[17]. Experimental evidence for phenotypic buffering of protein coding polymorphisms is well established [18],[19].

In fact, it has been shown that phenotypic buffering is a general property of complex gene-regulatory networks [20]. Also, if small heritable changes in transcript levels were transmitted unbuffered throughout the system, there would be a grave danger that genetic recombination would lead to unhealthy combinations of alleles and, consequently, to systems failure. Hotspots with large pleiotropic effects are thus more likely to be removed by purifying selection. If, as thus expected, common alleles are predominantly buffered by the robust properties of the system and hence largely inconsequential for the rest of the molecules in the system, this will have profound consequences for the design and interpretation of genetical genomics studies of complex diseases. Most importantly, it could turn out that even so-called common diseases—like diabetes, asthma, or rheumatoid arthritis—are not necessarily the result of common, small-effect variants in a large number of genes, but are rather caused by changes at a few crucial fragile points of the system (hotspots), which cause large, system-wide disturbances [21],[22]. Future studies in genetical genomics should aim at further elucidating the striking rarity of eQTL hotspots.
